# Toll-Like Receptor 7 Enhances Rabies Virus-Induced Humoral Immunity by Facilitating the Formation of Germinal Centers

**DOI:** 10.3389/fimmu.2019.00429

**Published:** 2019-03-08

**Authors:** Zhaochen Luo, Yingying Li, Ming Zhou, Lei Lv, Qiong Wu, Chen Chen, Yachun Zhang, Baokun Sui, Changchun Tu, Min Cui, Huanchun Chen, Zhen F. Fu, Ling Zhao

**Affiliations:** ^1^State Key Laboratory of Agricultural Microbiology, Huazhong Agricultural University, Wuhan, China; ^2^Key Laboratory of Preventive Veterinary Medicine in Hubei Province, College of Veterinary Medicine, Huazhong Agricultural University, Wuhan, China; ^3^Military Veterinary Research Institute, Academy of Military Medical Sciences, Changchun, China; ^4^Department of Pathology, University of Georgia, Athens, GA, United States

**Keywords:** rabies virus, Toll-like receptor 7, germinal center, humoral immunity, Th1

## Abstract

Rabies virus (RABV) causes fatal encephalitis in mammals and poses a public health threat in many parts of the world. Vaccination remains the most effective means for prevention and control of rabies. Studies focusing on the mechanism of RABV immunogenicity are necessary for improvement of rabies vaccines. Toll-like receptor 7 (TLR7), an innate receptor sensing single-stranded viral RNA, is important for the induction of innate and adaptive immunity. Our studies revealed that the absence of TLR7 led to a lower antibody production in mice immunized with RABV. It is further found that TLR7 deficiency affected the recruitment of germinal center (GC) B cells and led to lessened GCs formation. Consistently, there were less plasma cells (PCs) and antibody secreting cells (ASC) in TLR7^−/−^ mice than those in wild type (WT) mice, resulting in impaired production of RABV-neutralizing antibodies (VNA). TLR7 deficiency also impaired the generation of memory B cells (MBCs) and the induction of secondary immune responses. Moreover, TLR7 deficiency down-regulated the induction of some cytokines/chemokines, especially IFN-γ, resulting in a Th2-biased antibody production. Overall, our results suggest that TLR7 facilitates the induction of the humoral immunity in response to RABV.

## Introduction

Rabies causes fatal encephalitis in humans and other mammals, and continues to present a public health threat in many parts of the world. This disease causes more than 60,000 human deaths every year, most of which in developing countries (1). The etiological agent of rabies, RABV, belonging to the genus *Lyssavirus* within the family *Rhabdoviridae*, has a negative-sense single-stranded RNA (ssRNA) genome and five structure proteins (N, P, M, G, and L) (2). After biting by rabid animals, RABV enters into periphery neurons from the nearest neuromuscular junction (2). After a short incubation, RABV travels to the central nervous system (CNS) through motor neurons by retrograde axonal transport (3). Once RABV reaches the brain and replicates rapidly there, clinical symptoms will appear. Since there is no effective therapy available for rabies, vaccination is still the most effective method to prevent and control rabies (1).

The innate immune system is an important contributor to the activation and fine-tuning of adaptive immune responses (4). Upon virus infection, viral RNA is sensed by pattern recognition receptors (PRRs), which include Toll-like receptor 3 (TLR3), retinoid acid inducible gene-I (RIG-I), and melanoma-differentiation-associated gene-5 (MDA5), and/or TLR7 (5–7). Activation of these receptors leads to downstream signaling via TIR domain-containing adapter-inducing beta interferon (TRIF), interferon-promoter stimulator 1 (IPS-1), or myeloid differentiation primary response gene 88 (MyD88), respectively (8). The resulting signal cascade induces the production of type I interferon (IFN-α/β) and interleukins, which activate antigen-specific T and B cells (9). Of these PRRs, TLR7 plays an important role not only in the activation of the innate antiviral response, but also in the promotion of adaptive immunity, especially in the humoral immune responses (10–15).

In a previous report, TLR signaling on B cells initiates IgG1 and IgG2a/c class switching, whereas TLR-induced type I IFN production decreasing IgG1 and increasing IgG2a/c after immunization with influenza virus (10). In another study, Li et al. showed that TLR7 engagement is critical for the induction of RABV-specific antibody and Th1 bias in the first 10 days after intramuscular (i.m.) infection with SNBG, a RABV stain with a moderate pathogenicity, but the underlying mechanism remains unclear (7). In this study, we demonstrate that TLR7 is important for the induction of the humoral immune responses, especially the long-term immunity, by using an attenuated RABV strain. Furthermore, we found that TLR7 helps to recruit GC B cells and facilitates GC formation, which is critical for antibody production. Our findings pave the way for the development of novel RABV vaccines.

## Materials and Methods

### Viruses and Mice

LBNSE is a recombinant RABV derived from Street Alabama Dufferin (SAD)-B19, which is a widely used vaccine strain. Compared with the parental virus, LBNSE carries with two mutations in G protein at amino acid position 194 and 333 as described previously (16, 17). A wild-type RABV strain HuNPN01 isolated from rabid pigs in Hunan province, China, was used as a challenging virus after vaccination (18). TLR7^−/−^ (C57BL/6 genetic background; Stock No: 008380; 129S1- TLR7^tm1Flv^/J, USA) and TLR3^−/−^ (C57BL/6 genetic background; Stock No: 005217; 129S1- TLR3^tm1Flv^/J, USA) mice were obtained from Jackson Laboratories, and bred in the animal facility at Huazhong Agricultural University. Six-week-old C57BL/6 mice with same the gender to co-housed TLR7^−/−^ or TLR3^−/−^ mice were purchased from the Hubei Center for Disease Control, Wuhan, China.

### Flow Cytometry

Flow cytometry was conducted to quantify immune cells in the draining lymph nodes and bone marrow, as previously described (19, 20). Briefly, samples were collected and forced through a 40 μm nylon filter. Red blood cells were lysed with ACK lysis buffer (BioSource International, Inc., CA, USA), following the manufacturer's protocol. Single-cell suspensions (containing more than 10^6^ cells) were prepared in 0.2% bovine serum albumin (BSA) and stained for flow cytometric analysis. The antibodies used for flow cytometric analyses including FITC anti-mouse CD4 (clone GK1.5), APC anti-mouse CD185 (CXCR5) (clone L138D7), PE anti-mouse CD279 (PD-1) (clone 29F.1A12), FITC anti-mouse/human CD45R/B220 (clone RA3-6B2), APC/Cy7 anti-mouse CD19 Antibody (clone 6D5), APC anti-mouse CD69 Antibody (clone H1.2F3), Alexa Fluor 647 anti-mouse/human GL-7 (clone GL-7), PE anti-mouse CD95 (Fas) Antibody (clone SA367H8), APC anti-mouse CD138 (Syndecan-1) (clone 281-2), PE anti-mouse CD38 Antibody (clone 90) were all purchased from Biolegend, CA, USA. Data collection and analysis were performed using a BD FACSVerse flow cytometer (BD Biosciences, CA, USA) and FlowJo software (TreeStar, CA, USA).

### ELISA

CXCL13 (R&D Systems, MN, USA) ELISA kits were used according to the manufacturer's protocol. RABV-specific ELISAs were conducted to determine antibody isotypes as previously described (19, 21). Briefly, purified RABV virion proteins were used as immunoadsorbent. Bound Ab was detected using HRP-conjugated Abs (IgG (1:1,000), IgG1 (1:1,500), IgG2a (1:1,500), IgG2b (1:2,000), IgG3 (1:1,500), or IgM (1:2,000) (Boster, Wuhan, China). Color was developed using tetra-methyl-benzidine (TMB) substrate (Biotime Biotechnology, Shanghai, China), and reactions were stopped with 2M sulfuric acid. Optical densities were recorded at 450 nm using a SpectraMax 190 spectrophotometer (Molecular Devices, CA, USA).

### Histology and Immunofluorescence

Draining inguinal lymph nodes were fixed and dehydrated as previously described (20), flash frozen, and sectioned into 30 μm slices using a Microm HM 500M cryomicrotome (Thermo Fisher Scientific, Walldorf, Germany). Sections were incubated in Tris-buffered saline containing 5% BSA for 1 h at room temperature. GCs were visualized by labeling blocked sections with anti-CD45R/B220-Alexa Fluor 647 (Biolegend, CA, USA), anti-mouse IgG-Alexa Flour 488 (Biolegend, CA, USA), and anti-GL-7-streptavidin (eBioscience, CA, USA), followed by anti-biotin-Alexa Fluor 594 (Biolegend, CA, USA). The number of GCs was calculated according to the quantity of GL-7 positive cell clusters. All images were captured with a DP80/BX53 fluorescence microscope (Olympus, Tokyo, Japan).

### VNA Measurement

VNA titers were measured by using the fluorescent-antibody virus neutralization (FAVN) assay as previously described (20, 21). Briefly, serial dilutions of test serum and standard serum were prepared in 96-well micro plates. Each sample was added to four adjacent wells. A rabies challenge virus (CVS-11) suspension was added to each well, and the plates were incubated at 37°C for 1 h. Following incubation, suspended cells were added to each well, and the micro plates were incubated at 34°C in an incubator with 5% CO_2_ for 60 h. The samples were then fixed by the addition of 80% acetone for 30 min and air-dried. Cells were stained with FITC-conjugated antibodies against RABV N, and the results were assessed under an IX51 fluorescence microscope (Olympus, Tokyo, Japan). VNA titers were expressed in international units per ml by comparison with the titer of a reference serum obtained from the National Institute for Biological Standards and Control (Herts, United Kingdom).

### RNA-Seq Library Preparation, Sequencing, and Data Analysis

RNA-seq data was deposited in Gene Express Omnibus (GEO) with accession number GSE122637. Each sample was a mixture of draining lymph nodes from three mice and total RNA was extracted. RNA Nano 6000 Assay Kit of the Bioanalyzer 2100 system (Agilent Technologies, CA, USA) was used to evaluate RNA integrity. Only RNA samples that passed the quality tests were chosen for RNA-Seq analyses. RNA-Seq library construction was performed using the NEBNext Ultra Directional RNA Library Prep Kit for Illumina (NEB, MA, USA) following manufacturer's instructions and four index codes were added to attribute sequences to different samples. Products were purified with the AMPure XP beads system and quantified using the Agilent Bioanalyzer 2100 system. The clustering of the index-coded samples was performed on a cBot Cluster Generation System using the TruSeq PE Cluster Kit v3-cBot-HS (Illumina, CA, USA) according to the manufacturer's instructions. RNA-seq libraries were sequenced on an Illumina HiSeq X-Ten platform to generate 100 bp single-ended reads. Raw reads were pre-processed to remove low quality regions and adapter sequences. Read counts of each gene were summarized by the HTSeq-count30. The R package edgeR was used to identify the differentially expressed genes. The expression of each gene was normalized to reads per million (RPM) to compare among different samples. Lowly expressed genes were removed and only genes with an expression level of at least 1 RPM in at least two samples were kept for further analysis. Genes with fold change (FC) of +/−1.5 and an adjusted *P*-value (Padj) <0.05 were considered to be differentially expressed. Single differentially expressed genes were performed with Cluster 3 and Java TreeView.

### Quantitative Real Time PCR (qRT-PCR)

Total RNA was isolated by TRIZol Reagent (Invitrogen, Karlsruhe, Germany) and treated with DNase. RNA was then converted to cDNA by reverse transcription using FSQ-201 ReverTra Ace (TOYOBO, Osaka, Japan). Quantitative PCR analysis using SYBR green (BioRad, CA, USA) was performed on an Applied Biosystems 7300 Real-time PCR system (Applied Biosystems, CA, USA). Primer sets: FcGR1, Forward- AGGTTCCTCAATGCCAAGTG, Reverse- TGCCTGAGCAGTGGTAGATG; CCL2, Forward- GCTTCTGGGCCTGCTGTTCA, Reverse- AGCTCTCCAGCCTACTCATT; CCL4, Forward- CAGCCCTGATGCTTCTCACT, Reverse- GGGAGACACGCGTCCTATAAC; CXCL10, Forward- CCTGCTGGGTCTGAGTGGGA, Reverse- GATAGGCTCGCAGGGATGAT; IL6, Forward- ACAGAAGGAGTGGCTAAGGA, Reverse- CGCACTAGGTTTGCCGAGTA; IL27, Forward- ACTCTGCTTCCTCGCTACCA, Reverse- AGGGGCAGCTTCTTTTCTTC; IFN-γ, Forward- GACTGTGATTGCGGGGTTGT, Reverse- GGCCCGGAGTGTAGACATCT.

### ELISpot Assay

Commercial ELISpot kits (DAKEWE, Shenzhen, China) were used for measuring mouse IFN-γ and IL-4 secreting cell. Splenocytes were isolated at 7 days post infection (d.p.i.) and seeded into a 96-well plate. Cells were stimulated with inactivated and purified RABV virions and incubated in 5% CO_2_ at 37°C for 24 h. Plates were washed and processed according to the manufacturer's protocol. Plates were scanned and spots were quantitated. For B cells ELISpot, Multiscreen-HA ELISpot plates (Millipore, MA, USA) were coated with purified RABV virions and incubated for 16 h at 4°C. Coated plates were washed and blocked with RPMI 1640 supplemented with 10% FBS for 2 h in 37°C. Cell suspensions prepared from draining lymph nodes were transferred to the blocked ELISpot plates and conducted by using biotin conjugated mouse IgG antibody (Bethyl Laboratories, TX, USA), streptavidin-alkaline phosphatase (Mabtech, Stockholm, Sweden) and BCIP/NBT-plus (Mabtech, Stockholm, Sweden).

### Statistical Analysis

All data were analyzed using GraphPad Prism software (GraphPad Software, CA, USA). Significant differences between columns were determined by using Student's *t* test. The survival ratio was analyzed by Log-rank (Mantel-Cox) test. Asterisks in figures indicate statistical significance (^*^P < 0.05; ^**^P < 0.01; ^***^P < 0.001).

## Results

### TLR7 Is Important for Antibody Production After RABV Immunization

**Figure 1:**
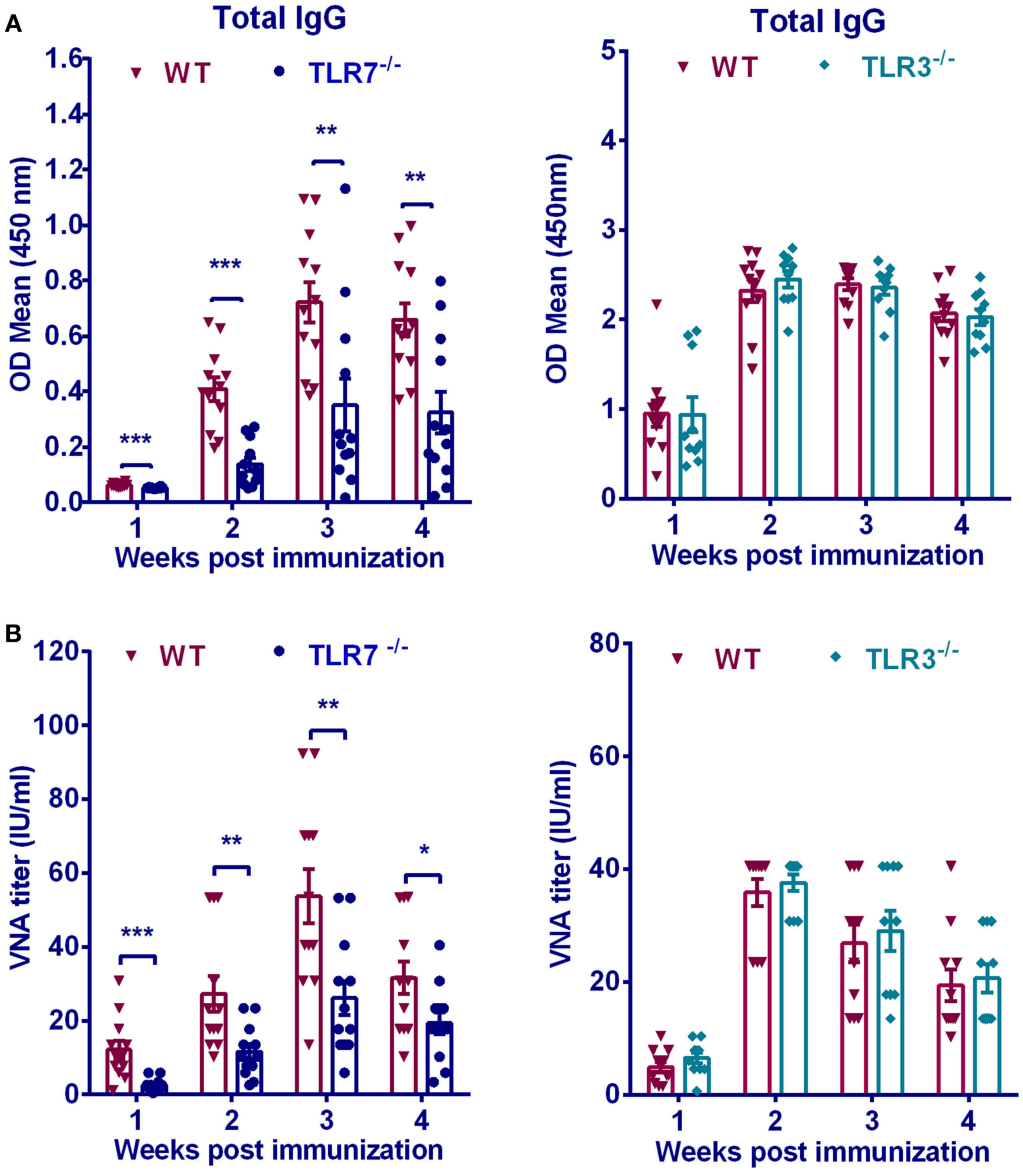
TLR7 is indispensable for optimal antibody production after RABV immunization. TLR7^−/−^(*n* = 12), TLR3^−/−^(*n* = 10), or WT mice (*n* = 11–12) were immunized i.m. with 10^6^ FFU RABV vaccine strain LBNSE in the hind legs. At indicated times post immunization, sera were collected for measuring RABV-specific total IgG **(A)** and VNA titers **(B)**. Error bars represent standard error of mean (SEM) (^*^P < 0.05; ^**^P < 0.01; ^***^P < 0.001).

To determine the contribution of TLR7 on humoral immunity, we measured RABV-specific antibody induced in TLR7^−/−^ and WT mice after RABV immunization. Previous studies showed that among TLR family TLR3 was found to be involved in RABV infection (5), thus its effect on humoral immunity after RABV vaccination was also evaluated. TLR7^−/−^, TLR3^−/−^ or WT mice were immunized intramuscularly with 10^6^ FFU RABV vaccine strain LBNSE. At indicated time points, blood was collected and antibody titers in the serum were measured. Total anti-RABV IgG levels in WT mice were 2 to 3-fold higher than those in TLR7^−/−^ mice at 2, 3, and 4 weeks post infection (w.p.i.) (Figure 1A). Consistently, WT mice maintained significantly higher levels of VNA titers than that in TLR7^−/−^ mice (Figure 1B). On the contrary, both total IgG and VNA levels in TLR3^−/−^ mice were not significantly affected compared with those in WT mice (Figures 1A,B). Taken together, these data demonstrated that TLR7 facilitates antibody production after RABV vaccination.

### TLR7 Deficiency Causes Defect in GC Formation After RABV Immunization

**Figure 2:**
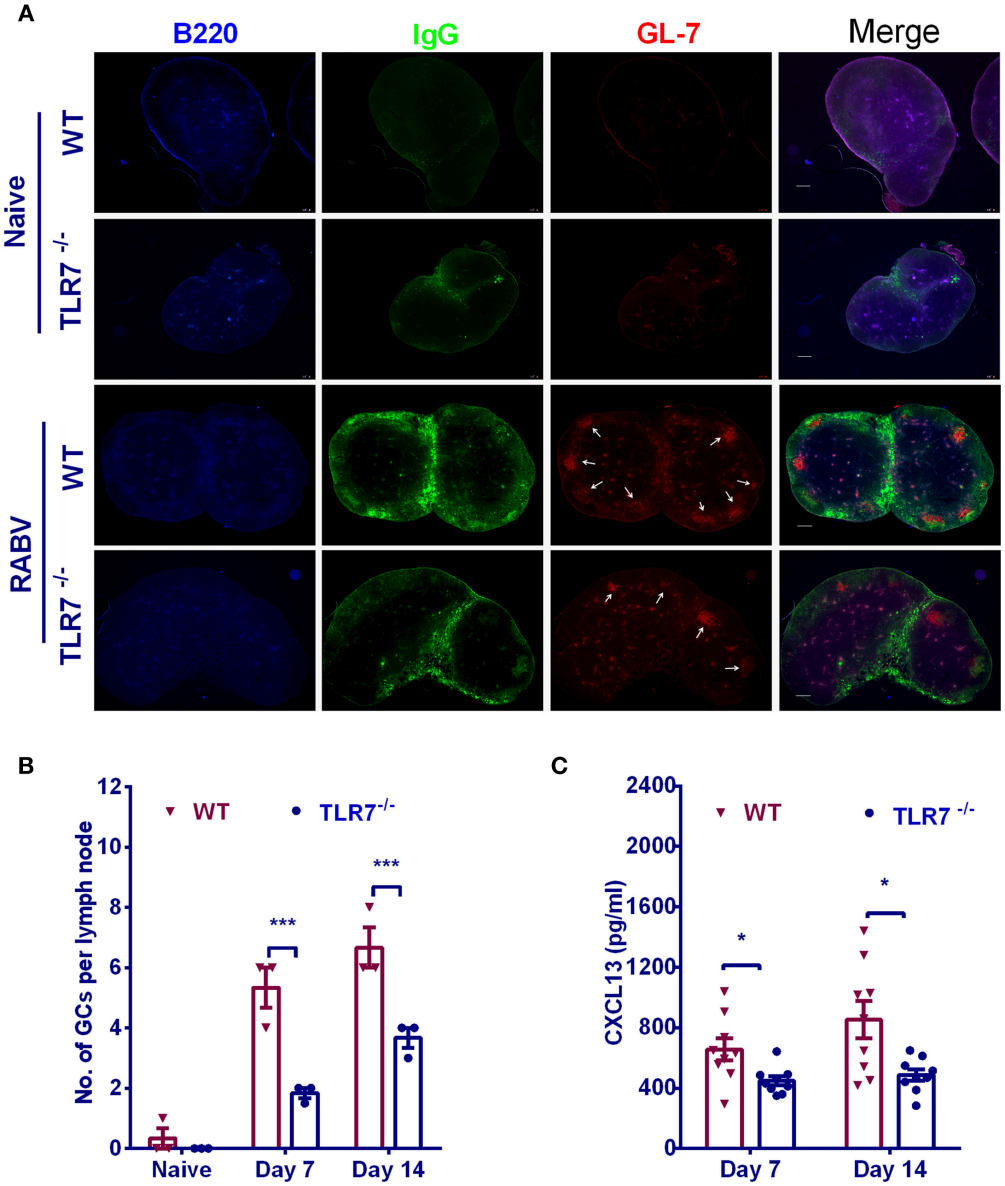
TLR7 facilitates the formation of GCs. **(A,B)** TLR7^−/−^ and WT mice were immunized i.m. with 10^6^ FFU LBNSE in the hind legs. Draining LNs were excised, and tissue sections were prepared and stained for GCs (GL-7, red; B220, blue; and IgG, green). Scale bars represent 200 μm. Representative sections are shown in **(A)** Numbers of GCs (GL-7 positive cell clusters labeled with white arrows) in the draining LNs are calculated and shown in **(B)** (*n* = 3). **(C)** Blood samples were collected at indicated time points and the concentration of serum CXCL13 was determined by using a commercial ELISA kit (*n* = 9). Error bars represent SEM (^*^P < 0.05; ^***^P < 0.001).

GCs are critical for the generation and selection of B cells that produce high-affinity antibodies. To determine whether TLR7 plays a role in the formation of GCs, we counted the number of GCs in inguinal LNs from TLR7^−/−^ and WT mice after immunization. There was significantly less in the number of GCs in TLR7^−/−^ mice than those in WT mice (Figures 2A,B). Since chemokine CXCL13 in serum is proved to be a biomarker of GC activity in human vaccine trials (22) and during RABV vaccination (20), we also analyzed plasma CXCL13 concentration in the first 2 weeks after immunization. Consistent with the results described above for GCs, a significantly lower level of CXCL13 was observed in TLR7^−/−^ mice (Figure 2C). Together, these data suggest that TLR7 is important for the formation of GCs.

### TLR7 Deficiency Limits GC B Recruitment After RABV Immunization

**Figure 3:**
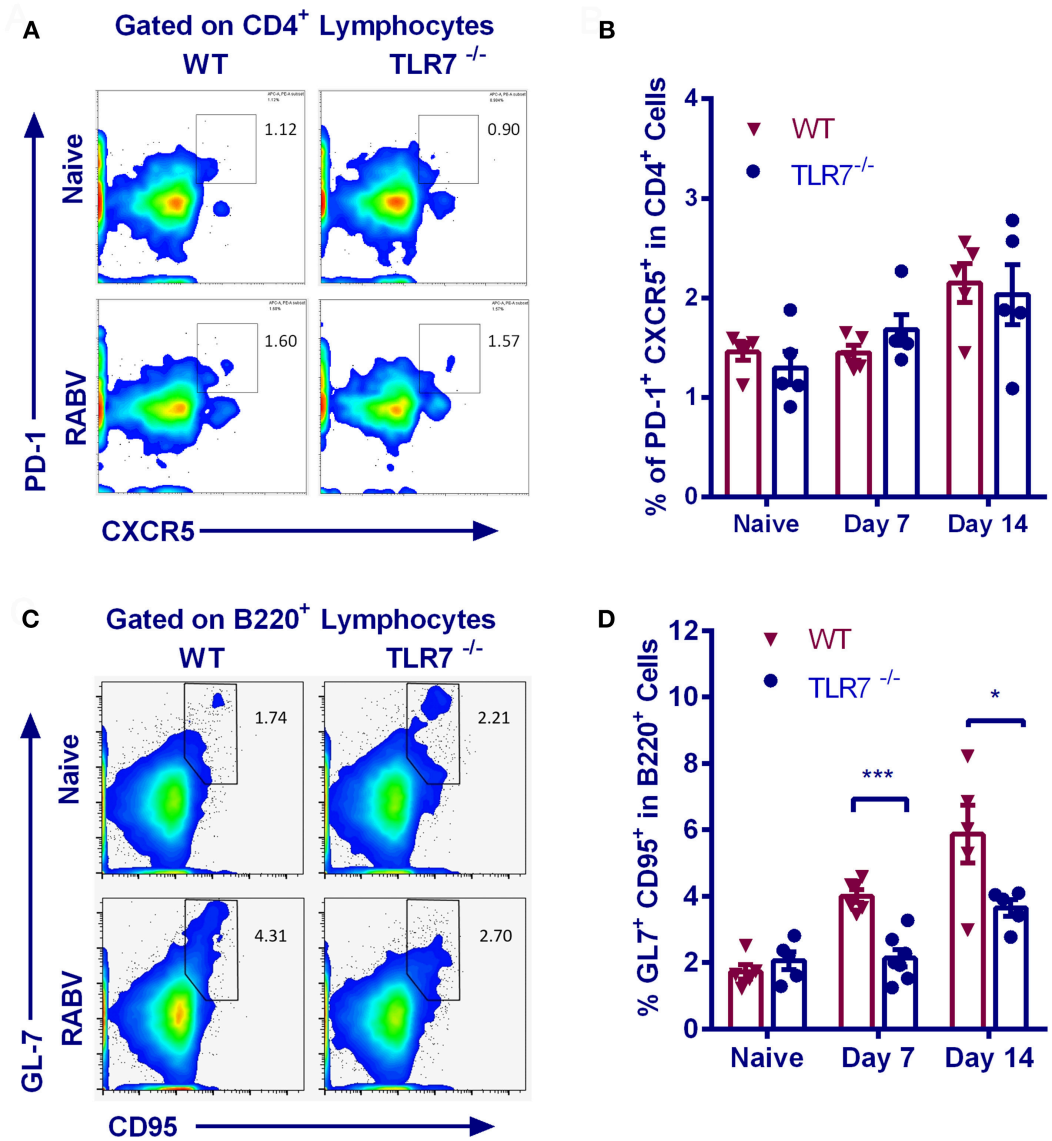
TLR7 deficiency limits GC B recruitment after RABV vaccination. TLR7^−/−^ and WT mice were immunized i.m. with 10^6^ FFU LBNSE in the hind legs. Draining lymph nodes were collected at the indicated times p.i. for analysis of Tfh cells (CD4^+^CXCR5^hi^PD1^hi^) and GC B cells (B220^+^GL-7^+^CD95^+^), and total 10^5^ events were acquired to generate the graphs. **(A,B)** Representative flow cytometry plots **(A)** and corresponding calculated frequencies **(B)** for Tfh cells (*n* = 5). **(C,D)** Representative flow cytometry plots **(C)** and corresponding calculated frequencies **(D)** for GC B cells (*n* = 5–7). Error bars represent SEM (^*^P < 0.05; ^***^P < 0.001).

Follicular helper T (Tfh) cells provide instructive signals that lead to the survival, affinity maturation, and fate decision of GC B cells (23, 24), which is important in the production of antigen-specific antibody. We therefore assessed the generation of Tfh cells (identified as CD4^+^CXCR5^hi^PD-1^hi^, Figure 3A) in TLR7^−/−^ and WT mice after RABV vaccination. However, frequency of Tfh cells in inguinal LNs from TLR7^−/−^ and WT mice at 7 and 14 d.p.i. was approximately equal (Figure 3B), suggesting that the generation of Tfh cells is not affected by TLR7 deficiency.

Naïve B cells can interact with viral antigens through B cell receptors, and then develop into GC B cells with the assistance of CD4^+^ T cells. Following stimulation, naïve B cells are recruited into the GC reaction and undergo iterative rounds of selection and proliferation, leading to the production of long-lived plasma cells (PCs) (25). We therefore analyzed the recruitment of GC B cells (B220^+^CD95^+^GL-7^+^) in inguinal LNs. Representative flow cytometric plots for GC B cells are shown in Figure 3C. GC B cells are equally abundant in TLR7^−/−^ and WT mice prior to vaccination, but their number in TLR7^−/−^ mice only increase to the levels about one half upon vaccination when compared to WT mice (Figure 3D).

### TLR7 Deficiency Impairs the Generation of PCs and ASCs

**Figure 4:**
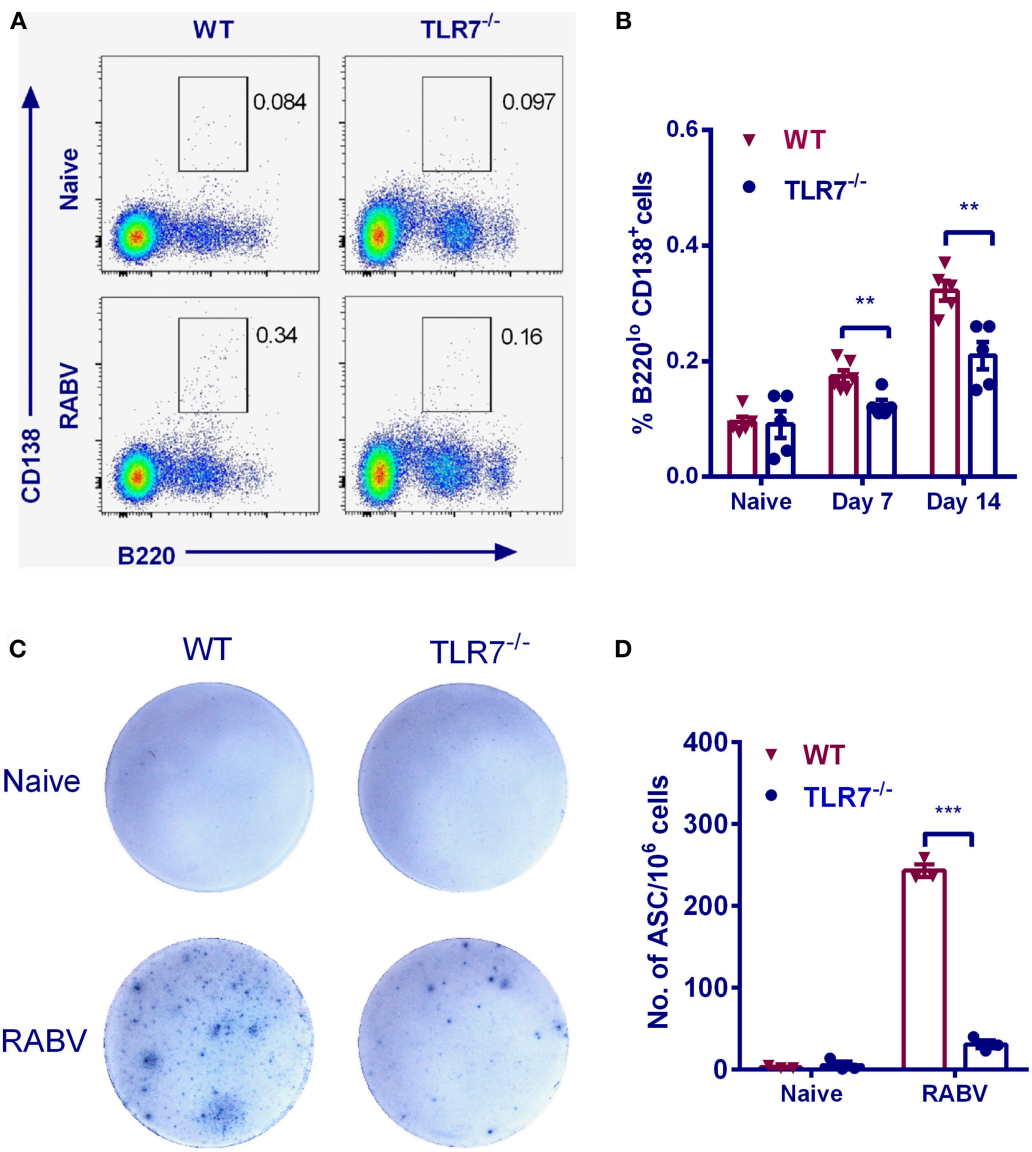
TLR7 promotes the generation of PCs and ASCs. TLR7^−/−^ and WT mice were immunized i.m. with 10^6^ FFU LBNSE in the hind legs. **(A,B)** Bone marrows were collected at the indicated times p.i. Total 10^5^ events were acquired for analysis of plasma cells (B220^lo^CD138^+^) (*n* = 5–6). **(C,D)** Draining lymph nodes were collected at 7 d.p.i. for analysis of RABV-specific ASCs by ELISpot (*n* = 3). Representative sections are shown in **(C)** and numbers of ASCs are shown in **(D)**. Error bars represent SEM (^**^P < 0.01; ^***^P < 0.001).

In the GC reaction, B cells that gain affinity for the cognate antigen expand preferentially (26). Eventually the GC is oligo-clonally populated with the progeny of these cells (27). During this process, some cells undergo differentiation to either PCs or MBCs via known and the unknown mechanisms (26). To examine the effects of TLR7 on PCs formation, the relative frequencies of PCs were assessed after RABV vaccination. The frequency of PCs (B220^lo^CD138^+^) in WT mice was significantly higher than that in TLR7^−/−^ mice by 7 and 14 d.p.i. (Figures 4A,B). Also, the generation of RABV-specific ASCs was evaluated by ELISpot assays. As expected, number of RABV-specific ASCs in TLR7^−/−^ mice did not increase to the same extent after immunization (about 8-fold lower) when compared with those in WT mice (Figures 4C,D). Together, these results indicate that TLR7 deficiency impairs the generation of PCs and ASCs after RABV immunization.

### TLR7 Deficiency Impairs the Production of Long-Lasting Antibodies

**Figure 5:**
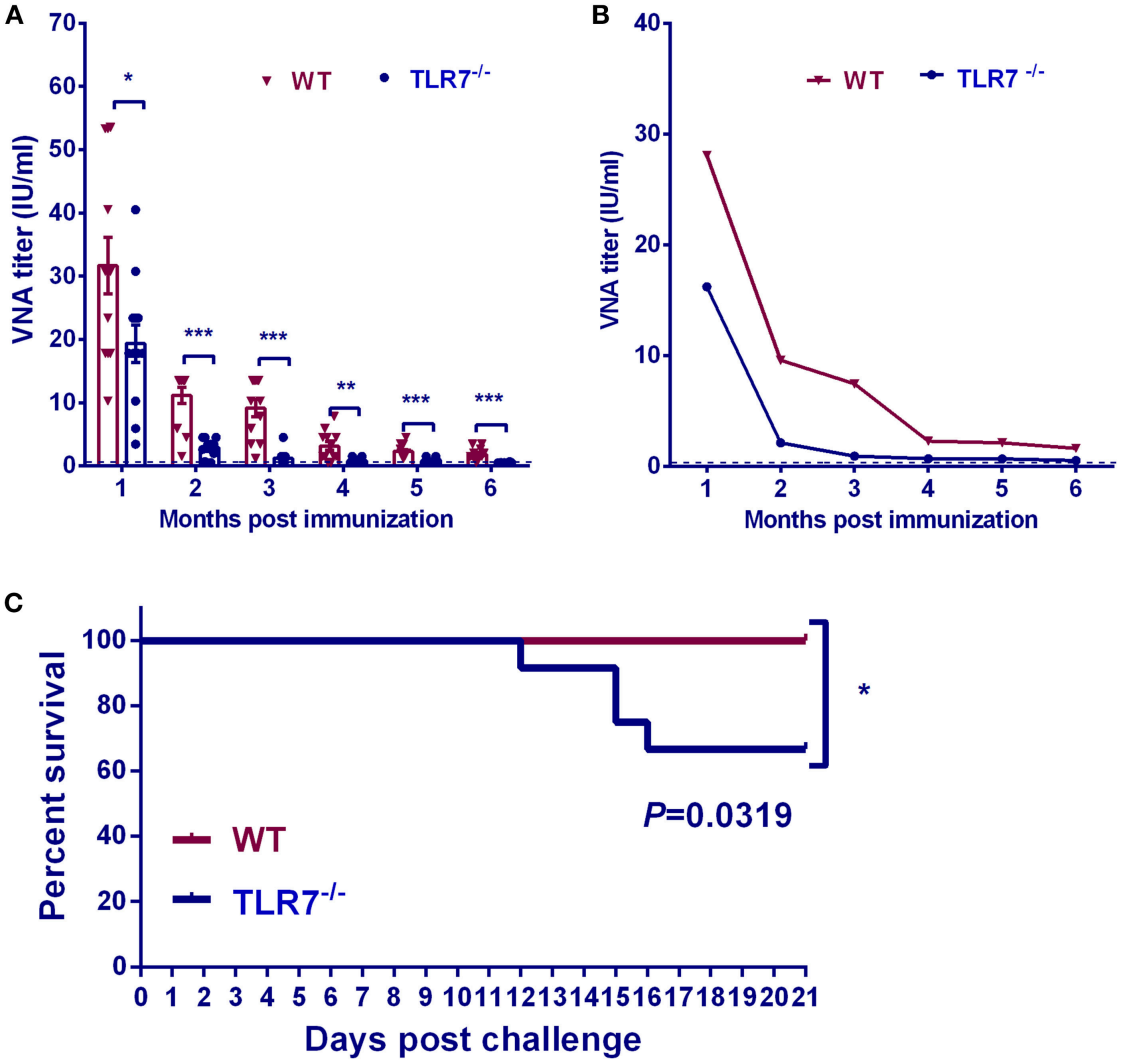
TLR7 helps to maintain a long-term antibody production. TLR7^−/−^ and WT mice were immunized i.m. with 10^6^ FFU LBNSE. At indicated time points, sera were collected for measurement of anti-RABV neutralizing antibody till 6 months post vaccination and results were expressed as arithmetic mean **(A)** and geometric mean **(B)** of VNA titers (*n* = 12). At 2 months post vaccination, mice were challenged with 50LD_50_ of a wild type RABV strain HuNPN01 and survival ratios were recorded in **(C)** (*n* = 12). Error bars represent SEM (^*^P < 0.05; ^**^P < 0.01; ^***^P < 0.001).

To identify the role of TLR7 on maintenance of antibody production in a long-term duration after RABV vaccination, mouse serum was collected and RABV-neutralizing antibodies (VNA) were measured at indicated time points for 6 months (Figure 5A). Geometric mean titer (GMT) of VNA was also calculated to display the kinetic of VNA production after RABV immunization (Figure 5B). Compared with TLR7^−/−^ mice, WT mice maintained significantly higher levels of VNA production at all the time-points after immunization. In addition, the role of TLR7 on long-term protection against lethal RABV challenge was investigated. At 2 months p.i, mice were challenged intramuscularly in the hind leg with 50 times 50% lethal doses (LD_50_) of a wild type RABV strain HuNPN01, and the survival ratio was monitored for another 3 weeks. Around 40% of the immunized TLR7^−/−^ mice succumbed to rabies within 16 days, while 100% of the immunized WT mice survived (Figure 5C). These data indicate that TLR7 helps to maintain the long-term antibody production and improve protection after RABV vaccination.

### TLR7 Helps to Improve the Secondary Antibody Responses

**Figure 6:**
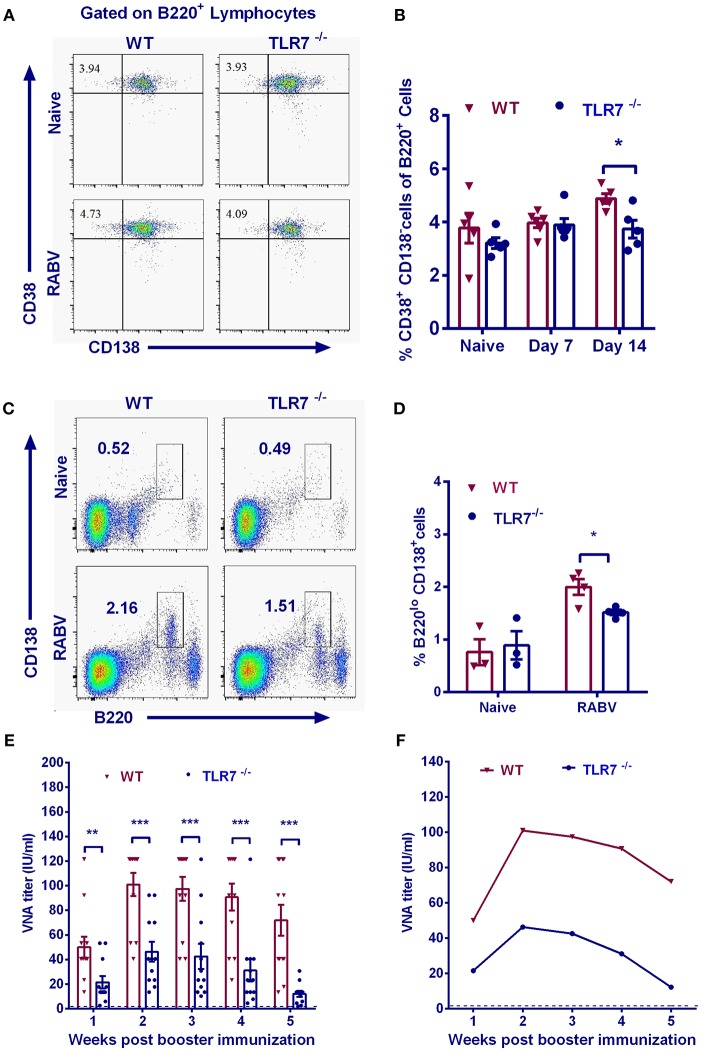
TLR7 augments MBCs generation and promotes secondary immune responses. TLR7^−/−^ and WT mice were immunized i.m. with 10^6^ FFU LBNSE in the hind legs. Draining lymph nodes were collected at indicated time points. Total 10^5^ events were acquired for quantification of MBCs (B220^+^CD38^+^CD138^−^). Representative flow cytometry plots **(A)** and corresponding calculated frequencies **(B)** are shown (*n* = 5–6). **(C–E)** 6 months after primary immunization, mice were boosted with 10^6^ FFU LBNSE. Bone marrow was collected for analysis of PCs (B220^lo^CD138^+^) at 14 d.p.i. **(C, D)** (*n* = 3–4). **(E,F)** At the indicated times after secondary immunization, blood samples were collected for measurement of VNA titers (*n* = 12). The arithmetic mean **(E)** and geometric mean **(F)** of VNA titers are calculated, respectively. Error bars represent SEM (^*^P < 0.05; ^**^P < 0.01; ^***^P < 0.001).

MBCs generate immunoglobulins rapidly and vigorously upon secondary infections (28). To assess whether TLR7 influences memory immunity after RABV immunization, we examined the abundance of MBCs by analyzing B220^+^CD38^+^CD138^−^ cells with flow cytometry after primary immunization. Significantly less MBCs were observed in TLR7 deficiency mice on 14 d.p.i. after the primary vaccination (Figures 6A,B).

To further investigate the effect of TLR7 on recall responses to RABV, TLR7^−/−^ and WT mice were boosted with 10^6^ FFU RABV at 24 weeks after primary vaccination. Two weeks after the secondary immunization, PCs (B220^lo^CD138^+^) in the bone marrow of WT mice were significantly more abundant than those in TLR7^−/−^ mice (Figures 6C,D). The arithmetic mean (Figure 6E) and geometric mean (Figure 6F) of the VNA titers in WT mice were much higher than that in TLR7^−/−^ mice after boost. Together, these results suggest that TLR7 is important for generation of MBCs and induction of secondary immune responses.

### RNA-Seq Analysis Reveals the Possible Immune Genes Regulated by TLR7

**Figure 7:**
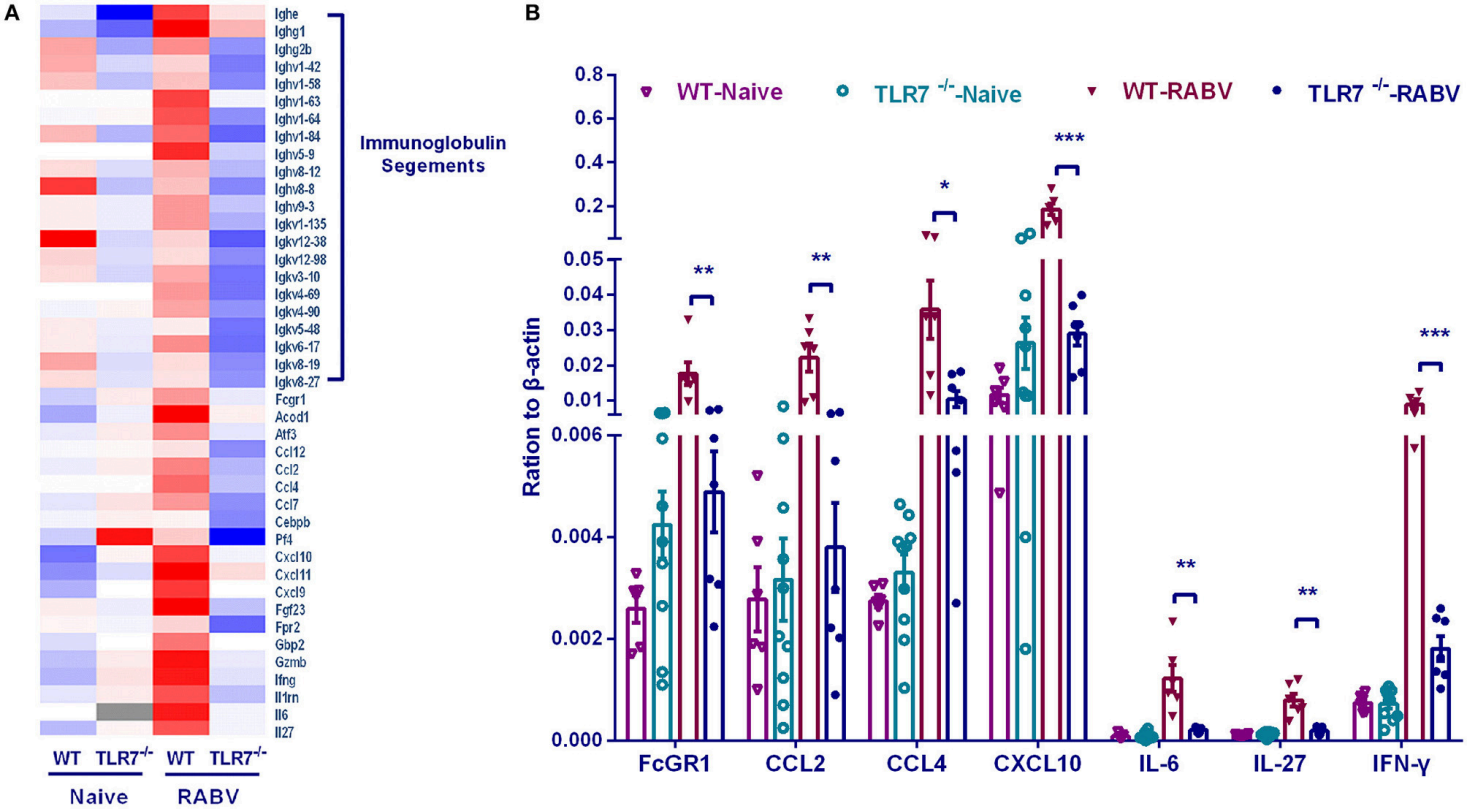
Gene transcription regulated by TLR7 after RABV vaccination. TLR7^−/−^ and WT mice were immunized i.m. with 10^6^ FFU LBNSE in the hind legs. Draining lymph nodes were collected at 7 d.p.i and the total RNA was isolated for RNA-seq. **(A)** After RNA-seq analysis, the representative differential genes are listed**. (B)** The transcriptional levels of selected differential genes were confirmed by qRT-PCR (*n* = 6–10). Error bars represent SEM (^*^P < 0.05; ^**^P < 0.01; ^***^P < 0.001).

To further investigate how TLR7 orchestrates humoral immune responses after RABV vaccination, we compared the transcriptome of draining lymph nodes from WT mice with that from TLR7^−/−^ mice by RNA-seq analysis. Individual gene analysis of differential genes identified some immunoglobulin segments and several cytokines (including IL-6, IL-27, IFN-γ, CCL2, CCL4, and CXCL10 etc.) were induced much more in WT mice than those in TLR7^−/−^ mice (Figure 7A). The up-regulation of some characterized cytokines (IL-6, IL-27, and IFN-γ) and chemokine (CCL2, CCL4, and CXCL10) were verified by qRT-PCR (Figure 7B).

### Absence of TLR7 Results in a Th2-Biased Immune Response

**Figure 8:**
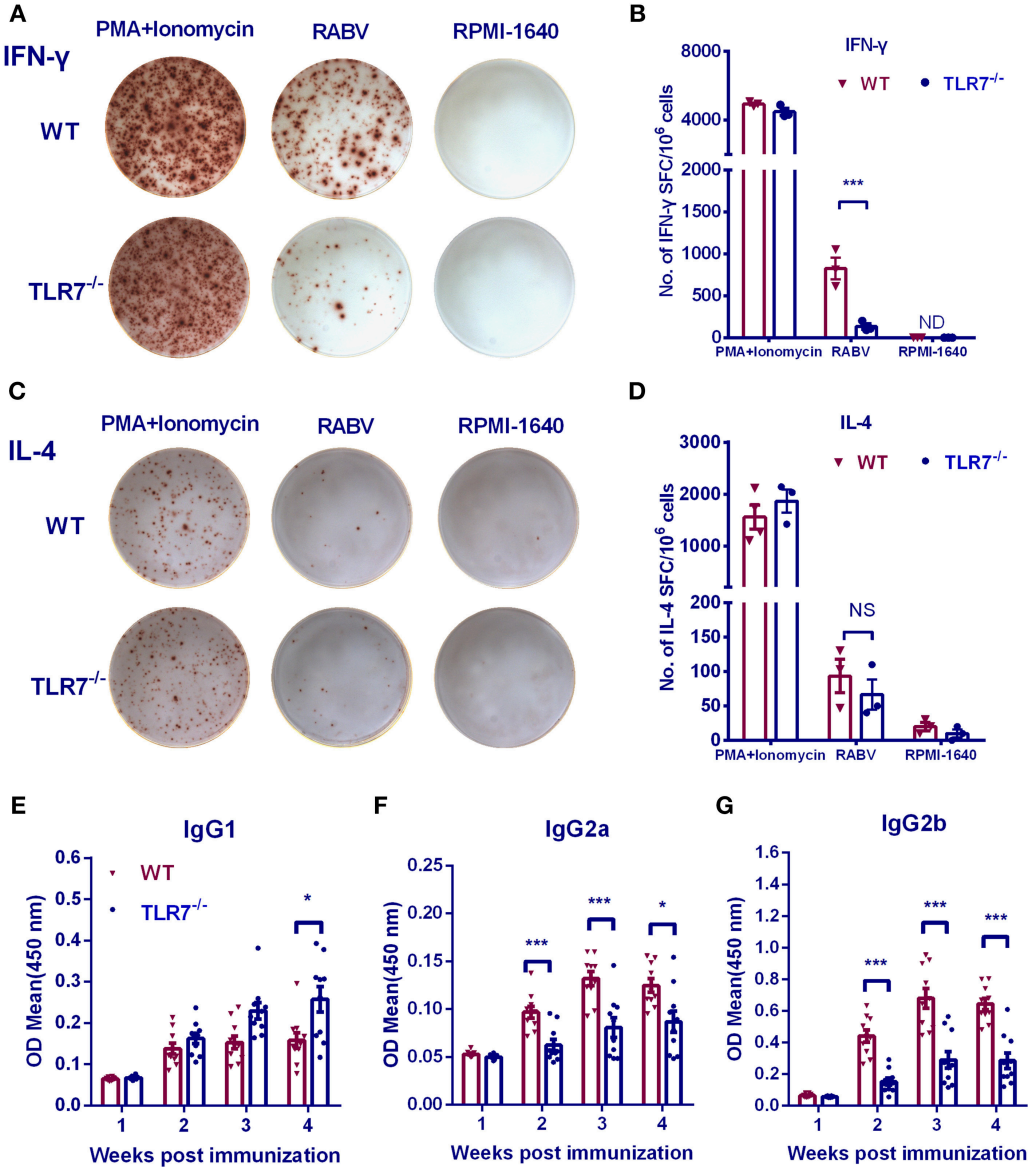
TLR7 facilitates Th1-biased immune responses after RABV vaccination. TLR7^−/−^ and WT mice were immunized i.m. with 10^6^ FFU LBNSE in the hind legs. Spleens were collected at 7 d.p.i. and subjected to ELISpot assay to measure RABV-specific IFN-γ **(A,B)** and IL-4 **(C,D)** producing cells (*n* = 3 mice/ group). **(E–G)** Blood samples were collected at the indicated times p.i. for analysis of RABV G-specific IgG1 **(E)**, IgG2a **(F)**, and IgG2b **(G)** (*n* = 10). Error bars represent SEM. ND, not detectable. NS, no significantly difference (^*^P < 0.05; ^***^P < 0.001).

RNA-seq analysis demonstrated that TLR7 up-regulated the production of cytokines and chemokines related to Th1 immune responses, especially IFN-γ, IL-6 IL-27, and CXCL-10. To determine whether TLR7 affects the immune polarization after RABV vaccination, splenic lymphocytes were collected from immunized mice, and ELISpot assays were performed. IFN-γ producing cells decreased about 6-fold in the absence of TLR7 (Figures 8A,B), while levels of IL-4 producing cells were not significantly affected (Figures 8C,D). We also compared IgG isotypes in TLR7^−/−^ and WT mice using ELISA at various times after primary immunization. RABV-specific IgG2a and IgG2b levels were significantly reduced in TLR7^−/−^ mice from 2 to 4 w.p.i. (Figures 8F,G). In contrast, significantly higher levels of IgG1 were observed in TLR7^−/−^ mice at 4 w.p.i. (Figure 8E). Together, these results demonstrate that TLR7 facilitates the generation of a Th1-biased immune response during RABV infection.

## Discussion

TLR7 binds single-stranded RNA, triggering a robust production of type-I IFN and inflammatory cytokines and thus limiting viral dissemination (29, 30). Besides its important role in innate recognition, TLR7 is also involved in adaptive immunity (4, 31). Li et al. found that TLR7 deficiency mice exhibited a phenotype with increased mortality with deficits in both the development of peripheral immunity and RABV clearance from the CNS (7). Recently, Liu et al. showed that a complex containing imiquimod, a TLR7 agonist, enhanced the immunogenicity of RABV vaccine by facilitating dendritic cells (DC) maturation as well as production of both type I interferon and pro-inflammatory cytokines (32). Above studies indicate the potential role of TLR7 on RABV induced humoral immunity.

The development of GC B cells in response to a pathogen is dependent on B cell-intrinsic MyD88 signaling (33). In experiments using CpG-NPCGG, a complex of TLR9 agonist and haptenated chicken gamma globulin, TLR9 was shown to boost affinity maturation and B-cell memory, and promote affinity maturation and B-cell selection in GCs (34). Here in the present study, by using a TLR7^−/−^ mouse model, we found that the absence of TLR7 significantly affects the formation of GC and the recruitment of GC B cells following RABV immunization.

To explore the underlying mechanisms, we analyzed the transcriptome in the lymph nodes of mice after RABV vaccination. Most of the differentially expressed genes were cytokines and immunoglobulin variable regions, while few surface molecules and transcriptional factors could be identified. Several cytokines/chemokines related to Th1 immune response, including IL-6, IFN-γ, IL-27, and CXCL10, were up-regulated in the attendance of TLR7 after RABV immunization. In the follicles, increased production of IL-6 by follicular dendritic cells (FDCs) may facilitate GC Tfh for maximal differentiation and function, which contributes to GC B cells differentiation, although no improvement in total Tfh differentiation was found (23). However, we found that TLR7 does not affect Tfh generation, in agreement with Clingan's finding that Tfh cell differentiation is not impaired in the absence of TLR7 (35). Interestingly, Wu and colleagues found that the absence of FDC-IL-6 correlated with a reduction in somatic hyper-mutation (SHM) that coincided with the reduction in GCs and antigen-specific antibodies, suggesting that FDC-derived IL-6 is physiologically relevant in generating GC reactions, SHM and IgG production (36).

IFN-γ is secreted by T helper cells (specifically, Th1 cells), cytotoxic T cells, macrophages, mucosal epithelial cells and NK cells, causing undifferentiated CD4^+^ cells (Th0 cells) to differentiate into Th1 cells and contributing to the generation of a Th1-biased immune response (37). IL-27 plays an important role in the early stages of Th1 commitment, favoring a Th1 response (38). CXCL10 is reported to facilitate the interaction of T cells with DC or B cells (39). Mature DC-derived CXCL10 is pivotal to retain Th1 lymphocytes within T cell areas of the draining LNs and optimize the Th1-mediated immune responses (7, 40). These cytokines/chemokines may facilitate the induction of Th1-baised immune responses in WT mice compared with TLR7^−/−^ mice.

We subsequently demonstrated an inadequate Th1-biased immune response was observed in TLR7^−/−^ mice, suggesting that TLR7 is critical for Th1 polarization following RABV immunization. Hooper et al. found that Th1 cells are critical in the clearance of RABV from CNS (41, 42). Activation of the TLR7/9 signaling pathway in T cells leads to T-bet expression in B cells and improves IgG2a isotype switching, and vice versa (43–45). Besides, TLR7 stimulation may activate the infiltrating myeloid-derived suppressor cells (MDSC) to further differentiate into other activated myeloid cell types and prevent the suppression of Th1 polarized immune responses (15).

## Conclusion

We demonstrate that TLR7 contributes to RABV-induced antibody production by facilitating the formation of GCs and the recruitment of GC B cells. Further studies showed that TLR7 up-regulated some cytokines and chemokines expression, resulting in a Th1-baised antibody production. Our results highlight the role played by TLR7 in inducing humoral immunity, especially the long-term immunity, after RABV vaccination. Targeting the TLR7 signaling pathway for activation is a promising strategy for improving the efficiency of rabies vaccines.

## Data Availability

RNA-seq data has been deposited on NCBI Gene Expression Omnibus and are accessible through GEO accession number GSE122637.

## Ethics Statement

All animals involved in the study were housed and handled according to recommendations in the Guide for the Care and Use of Laboratory Animals of the Ministry of Science and Technology of China and protocols approved by the Scientific Ethics Committee of Huazhong Agricultural University (permit number HZAUMO-2015-023).

## Author Contributions

ZL and LZ conceived and designed the experiments. ZL, YL, LL, QW, and CC performed the experiments. ZL, MZ, YZ, BS, CT, MC, HC, ZF, and LZ analyzed the data. ZL, MZ, ZF, and LZ wrote the article.

### Conflict of Interest Statement

The authors declare that the research was conducted in the absence of any commercial or financial relationships that could be construed as a potential conflict of interest.
